# Identification of key ferroptosis-related targets in colorectal cancer: A transcriptomics-driven study via machine learning and AUcell analysis of single-cell RNA-sequencing

**DOI:** 10.7150/jca.114522

**Published:** 2026-01-01

**Authors:** Zhiqiang Liang, Zehui Hou, Zhuomin Yu, Bing Zeng, Fang Li, Jingjing Wu, Yingru Li, Zhipeng Jiang

**Affiliations:** 1 Department of General Surgery, Hernia and Abdominal Wall Surgery, Guangdong Provincial Key Laboratory of Colorectal and Pelvic Floor Diseases, Biomedical Innovation Center, The Sixth Affiliated Hospital, Sun Yat-sen University, Guangzhou510655, Guangdong Province, China.; 2 Division of Gastrointestinal Surgery, Department of General Surgery, Shenzhen People's Hospital (The Second Clinical Medical College, Jinan University; The First Affiliated Hospital, Southern University of Science and Technology), Shenzhen 518020, Guangdong Province, China.

**Keywords:** ferroptosis, colorectal cancer, machine learning, bioinformatics, AUcell analysis

## Abstract

**Background:** Colorectal cancer (CRC) has emerged as the third most prevalent malignancy worldwide. The pursuit of dependable molecular signatures stands as a crucial endeavor for tailoring treatment approaches, refining prognostic assessments, and heightening therapeutic efficacy in CRC management. This investigation was conducted to elucidate essential genes and molecular mechanisms associated with ferroptosis in CRC through implementing machine-learning approaches and AUcell analysis.

**Methods:** The GEO repository and FerrDb served as primary sources for extracting information of gene sets on colorectal cancer and iron-dependent cell death mechanisms. To determine potential therapeutic targets with biomarker significance, we implemented LASSO and SVM-RFE methodology. The immune infiltrates were characterized followed by a competing endogenous RNA network analysis. The AUCell R package was utilized to examine the targeted gene activity patterns within individual cell lines using single-cell transcriptome data. The qRT-PCR and Human Protein Atlas (THPA) database were used to validate the expression of target genes. Potential therapeutic were explored through the DGIdb database.

**Results:** Through the application of machine learning methodologies, five genes were identified as pivotal biomarker candidates: AQP8, NOX4, NR5A2, SCD, and TIMP1. The result of AUcell algorithm showed that the distribution of AUC values exhibited a bimodal pattern, with 2733 cells demonstrating elevated AUC values above the threshold of 0.091. The result of qRT-PCR showed that NOX4, SCD, and TIMP1 were significantly upregulated, while the expression of AQP8 and NR5A2 did not exhibit the expected differences. Both mRNA and IHC analyses from HPA database confirmed the abnormal expression of these pivotal candidate biomarkers. Algorithmic assessment via CIBERSORT methodology revealed notable shifts in immune cell composition within the tumor microenvironment of individuals diagnosed with CRC. Furthermore, A competing endogenous RNA network and 51 potential drug candidates were identified.

**Conclusion:** A systematic framework implementing machine-learning approaches and AUcell analysis was established for identifying core ferroptosis genes and validating their functional link to ferroptosis. Meanwhile, a reliable ferroptosis-associated signature was established, which shed new light on the ferroptosis-mediated molecular mechanisms and therapeutic potential underlying CRC.

## 1. Introduction

Colorectal cancer (CRC) has emerged as the third most prevalent malignancy worldwide and ranks second in cancer-related mortality, with statistics indicating approximately 1.9 million new diagnoses and 903,859 fatalities in 2022^1^. Despite recent improvements in mortality and morbidity rates through enhanced endoscopic screening protocols, recurrence and metastasis continue to pose significant clinical challenges^2^. The prognosis for metastatic CRC remains poor, with five-year survival rates hovering around 20%^3^. Contemporary therapeutic approaches for CRC encompass surgical intervention, chemotherapeutic regimens, and radiation therapy. The advent of immunotherapeutic strategies, including anti-PD-1, anti-PD-L1, and anti-CTLA4 treatments, has revolutionized the treatment landscape for CRC, demonstrating remarkable therapeutic potential^4^. Novel combinations of radiotherapy and immunotherapy have been documented to elicit potent antitumor immune responses^5^, ^6^. However, chemoresistance persists as a fundamental obstacle in managing metastatic disease, ultimately contributing to treatment failure^7^. Furthermore, current methodologies are constrained by tumor heterogeneity, limited precision, and inadequate representation of diverse patient populations^8^, ^9^. The pursuit of dependable molecular signatures stands as a crucial endeavor for tailoring treatment approaches, refining prognostic assessments, and heightening therapeutic efficacy in CRC management. Identifying such specific biomarkers remains essential within the scope of precision medicine, particularly when developing customized intervention protocols for individual patients diagnosed with this digestive tract malignancy.

Ferroptosis, a distinct form of programmed cell death, is notable for iron dependency and lipid peroxide accumulation in cellular membranes. This process is regulated by both biochemical and genetic factors. Its significance extends beyond cellular death, playing crucial roles in tumor progression and therapeutic responsiveness across diverse malignancies, often interacting with reactive oxygen species in cancer-related pathways^9^. The hallmarks of ferroptosis include mitochondrial morphological alterations, iron accumulation, reactive lipid oxygen species generation, and the activation of specific genetic pathways^10^, ^11^. Emerging evidence suggests that ferroptosis induction in CRC cells may represent a promising therapeutic strategy^12^, ^13^. Consequently, the identification and characterization of ferroptosis-related genes in CRC are essential for advancing our understanding of disease pathogenesis, improving therapeutic approaches, and enhancing prognostic accuracy.

This research employs sophisticated machine learning algorithms and AUcell analysis methods to identify and characterize ferroptosis-associated genes in CRC. These findings offer fresh insights into the ferroptosis-associated molecular mechanisms underlying CRC and potential therapeutic interventions targeting these genes.

## 2. Materials and Methods

### 2.1 Data collection and processing

The experimental workflow is depicted in Supplementary Fig. S1. Expression profiles of CRC and normal tissue samples were acquired from the Gene Expression Omnibus (GEO) and The Cancer Genome Atlas (TCGA) databases [https://www.ncbi.nlm.nih.gov/geo/ (GEO); https://portal.gdc.cancer.gov/databases (TCGA)]^14^, ^15^. The dataset was partitioned into training and validation cohorts. The training set comprised GSE44861 (55 normal, 56 CRC samples), GSE110225 (30 normal, 30 CRC samples), and GSE113513 (14 normal, 14 CRC samples) (detailed in Supplementary Table S1). Batch effects were eliminated using the "sva" R package. For validation, GSE106582 (117 normal, 77 CRC samples), GSE161277 single cell RNA Sequencing data (3 normal, 4 CRC samples), and the Cancer Genome Atlas Colon Adenocarcinoma data (TCGA-COAD) was employed. Clinical annotations were extracted from TCGA-COAD. A comprehensive set of ferroptosis-related genes (FRGs; n = 563) was retrieved from FerrDb (detailed in Table S2). Potential drug-gene interactions were systematically examined utilizing the Drug-Gene Interaction Database (DGIdb).

### 2.2 Differential expression analysis

Expression data for 363 FRGs was extracted from the training cohort (representing all expressed FRGs in the training set; Table S3). Differential expression between tumor and normal samples was determined using the “Limma” R package. Significance thresholds were set at fold change |logFC| > 0.5 and adjusted P-value < 0.05.

### 2.3 Functional enrichment analysis

The clusterProfiler R package was employed to conduct Gene Ontology (GO) and Kyoto Encyclopedia of Genes and Genomes (KEGG) pathway analyses on the identified differentially expressed FRGs, with statistical significance threshold established at P < 0.05 (as detailed in Table S4). For graphical representation of these findings, the ggplot2 package was utilized. To gain deeper insights into the biological relevance of the genes, we implemented Gene Set Enrichment Analysis (GSEA), a computational methodology that evaluates whether predefined gene collections exhibit statistically significant concordant differences between biological states. This analysis was executed using the official GSEA software platform (https://www.gsea-msigdb.org/gsea/index.jsp) maintaining a significance criterion of P < 0.05 throughout the evaluation process (as detailed in Table S5).

### 2.4 Identification and selection of characteristic biomarkers through machine learning approaches

Two distinct machine learning methodologies were employed to identify ferroptosis-related genes (FRGs) linked to CRC: the Least Absolute Shrinkage and Selection Operator (LASSO), Support Vector Machine Recursive Feature Elimination (SVM-RFE). The LASSO algorithm was implemented via the glmnet package^16^ to perform dimensionality reduction, whereby differentially expressed FRGs between CRC patients and normal samples were selected. Subsequently, the SVM-RFE model was was applied to identify optimal variables through the elimination of SVM-generated eigenvectors. Hyperparameter optimization was conducted using the 'Caret' package, employing a grid search method with 10-fold cross-validation on the training dataset. The diagnostic significance of biomarkers exhibiting enhanced discriminative capacity was determined using the 'e1071'package^17^. The representative genes were determined through the intersection of genes identified by all two methodological approaches, resulting in the identification of five distinctive genes.

The predictive capability of these optimal gene biomarkers was evaluated through generation of Receiver Operating Characteristic (ROC) curves and the evaluation of critical performance metrics, involvingarea under the curve (AUC), accuracy, sensitivity, and specificity. A logistic regression model was constructed using the glm function in R, based on the five identified marker genes, enabling sample type predictions in both the GSE106582 and TCGA-COAD datasets. The model's predictive performance was subsequently evaluated using ROC curve analysis.

### 2.5 Single-cell transcriptome analysis pipeline

The raw data from GSE161277 was retrieved from GEO databases. Single-cell transcriptome analysis was performed utilizing Seurat v4.1.0 (http://satijalab.org/seurat/)^18^ in R, encompassing normalization, scaling, and cell clustering procedures, which ultimately identified 8 distinct cell types. Cell filtration was implemented using stringent quality control parameters (nFeature_RNA > 200, percent.mt < 10%, nCount_RNA > 1000). Doublets and non-viable cells were eliminated through the application of DoubletFinder package (version 2.0.2)^19^ and Scrublet v0.2.1^20^. The filtered gene-barcode matrices underwent normalization via the 'LogNormalize' method Following scaling, the 'FindVariableFeatures' function employing the 'vst' method was utilized to identify 2000 highly variable genes. Dimensionality reduction was achieved through principal component analysis (PCA) on a basis of these variable genes, with batch effects being mitigated using the Harmony package^21^. The resulting dimensionality-reduced clusters were visualized on the 2D map generated through t-distributed Stochastic Neighbor Embedding (t-SNE) utilizing Seurat's 'FindNeighbors', 'FindClusters' and 'runTSNE'. Differential gene expression analysis was conducted using the Kruskal-Wallis test.

### 2.6 Differential expression analysis and ferroptosis-related gene scoring

Differentially expressed genes (DEGs) within each cluster were examined to identify DE-FRGs based on the previously established characteristic biomarkers. Five DE-FRGs within the DEGs were selected from the DEG pool for scoring using AUCell (Version 1.12.0)^22^. The AUCell R package implemented pathway scoring for individual cells based on gene set enrichment analysis principles. Cell-specific gene expression rankings were generated according to the AUC values of the selected DE-FRGs, quantifying the proportion of highly expressed genes within each cell. Higher AUC values were indicative of increased gene set expression. The "AUCell_exploreThresholds" function was employed to establish thresholds for identifying cells with active gene sets. Subsequently, cellular AUC scores were projected onto the UMAP embedding and visualized using the ggplot2 R package to highlight active clusters.

### 2.7 Cell lines

CRC cell line (HCT116) and normal colorectal epithelial cell line (NCM460) were purchased from Guangzhou Cellcook Biotech Co., Ltd (Guangzhou, China). These cells were cultured in RPMI-1640 or DMEM medium supplemented with 10% FBS, maintained at 37 °C in a humidified incubator with 5% CO₂, and subcultured when reaching 70-90% confluency according to the supplier's instructions.

### 2.8 RNA extraction and PCR

Total RNA was extracted using Trizol (servicebio technology CO., LTD., Wuhan, China) and reverse-transcribed into cDNA using the PrimeScript RT reagent Kit (TaKaRa Biotechnology). The Applied Biosystems 7500 Sequence Detection system was used to perform quantitative real-time reverse transcription PCR (qRT-PCR) with SYBR Green PCR Master Mix (Applied Biosystems, Foster City, CA, USA). Each experiment was repeated independently at least thrice. The relative mRNA expression levels of the target genes were calculated using the 2^-∆∆Cq^ method and normalized to that of GAPDH. All the primer sequences are listed in Table S11. Statistical analyses were performed by two-tailed Student's t-test using Graphpad Prism (version 10.1.2).

### 2.9 The Human Protein Atlas (HPA) database

The Human Protein Atlas (HPA; https://www.proteinatlas.org) is a public database that enables validation of target gene expression. It characterizes protein expression in 44 major human tissues and several cancer types via immunohistochemistry^23^, with all images and annotations available for download. In the present study, we compared the mRNA expression levels (Table S12) and immunohistochemical (IHC) staining patterns of five pivotal ferroptosis-associated genes between human normal colorectal tissues and colorectal cancer tissues. Statistical analyses were performed using GraphPad Prism (version 10.1.2), with p-values determined via the Mann-Whitney U-test.

### 2.10 Analysis of immune cell infiltration

CIBERSORT methodology was implemented to characterize tissue cellular composition based on gene expression profiles^24^. Utilizing the Training dataset, predictive analysis was conducted to estimate the relative abundance of 22 distinct infiltrating immune cell populations within each tissue specimen (Table S6). Cumulative scores for all evaluated immune cell types were calculated per sample^25^. This analysis was executed using CIBERSORT package in R Studio, with only samples yielding CIBERSORT outputs of P < 0.05 being selected for further investigation. The statistical relationship between candidate biomarkers and immune cells exhibiting significant alterations was assessed by calculating Spearman correlation coefficients. This non-parametric analysis was executed through computational implementation utilizing the 'reshape2' and 'ggExtra' packages within the R programming environment.

### 2.11 CeRNA network

The construction of mRNA-miRNA interaction pairs was accomplished through the integration of three databases: miRanda, RNAhybrid, and PITA, utilizing the five identified marker genes. Interactions consistently predicted across all three databases were selected for subsequent analysis. The predicted miRNAs were then cross-referenced with the Ensembl database, and miRNA-lncRNA pairs were filtered to establish a comprehensive ceRNA network encompassingmRNA-miRNA-lncRNA interactions.

## 3. Results

### 3.1 Screening of DE-FRGs betwween the CRC and normal samples

**Figure 1:**
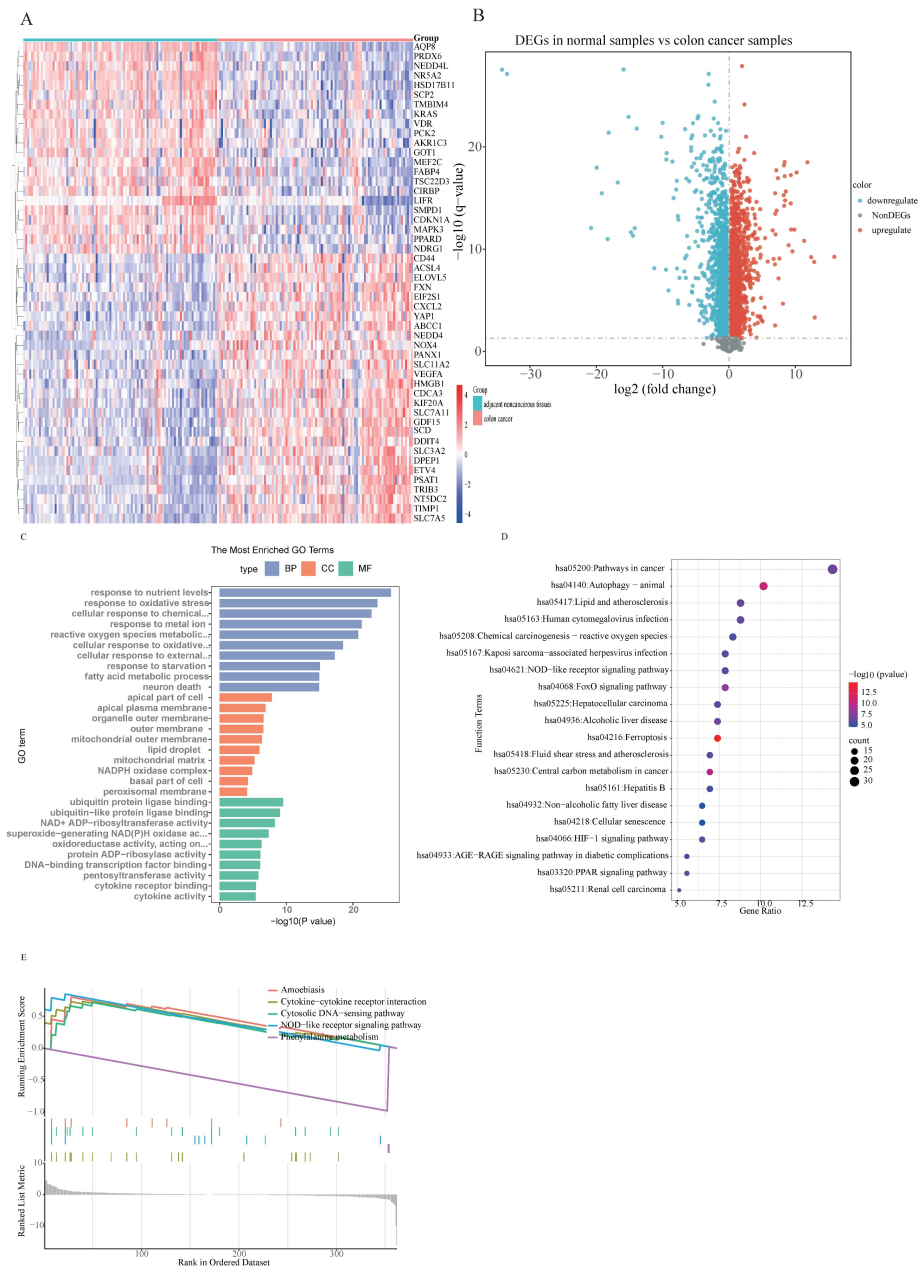
The expression levels and functional analysis results of ferroptosis-related genes in CRC: **(A)** A heatmap illustrating the expression patterns of DE-FRGs across the samples (Heatmap displaying the top fifty DEGs.); **(B)** Volcano plot of DEGs constructed using the fold change values and P-adjust;** (C)** Gene Ontology (GO) enrichment analysis reveals significant associations of DE-FRGs with functions such as “nutrient levels,” “ oxidative stress responses,” “ chemical stress,” and “ metal ion, ”; **(D)** Kyoto Encyclopedia of Genes and Genomes (KEGG) enrichment analysis highlights significant associations between DE-FRGs and pathways related to “autophagy,” “ferroptosis,” “Central carbon metabolism,” and “FoxO signaling pathway,”; **(E)** GSEA-KEGG pathway analysis significant associations between DE-FRGs and pathways related to “Cytokine-cytokine receptor interaction,” “Phenylalanine metabolism,” “NOD-like receptor signaling pathway,” “Cytosolic DNA-sensing pathway,” and “Amoebiasis”.

Differential expression analysis was conducted on 100 CRC samples and 99 normal samples within the merged dataset (comprising GSE44861, GSE110225, and GSE113513) utilizing the 'sva' R package. From the 363 ferroptosis-related genes (FRGs) examined, 217 exhibited significant expression disparities between CRC and normal samples, consisting of 121 upregulated and 96 downregulated genes (Fig. 1B), as identified from the merged data cohort (Table S7). The distribution and visualization of these differentially expressed genes are depicted in the heatmap and volcano plot presented in Fig. 1.

### 3.2 Functional enrichment analysis of DE-FRGs' signaling pathways

In our investigation into the underlying molecular mechanisms of colorectal carcinoma, a thorough analytical investigation was conducted on the identified ferroptosis-related genes showing differential expression patterns, including GO analysis, KEGG analysis and GSEA analysis. The outcomes of the GO functional enrichment analysis are demonstrated in Fig. 1C. Regarding biological processes, DE-FRGs were determined to be implicated in cellular response to nutrient levels, oxidative stress responses, chemical stress, metal ion, reactive oxygen species metabolic process, starvation, external stimulus, and fatty acid metabolism. Alterations in cellular components were predominantly correlated with apical part of cells, apical plasma membrane, organelle outer membrane, outer membrane, mitochondrial outer membrane, lipid droplet, mitochondrial matrix, and NADPH oxidase complex. Concerning molecular functions, DE-FRGs were characterized by activities related to ubiquitin protein ligase binding, ubiquitin-like protein ligase binding, NAD+ ADP-ribosyltransferase activity, superoxide-generating NAD(P)H oxidase activity, oxidoreductase activity, protein ADP-ribosylase activity and cytokine receptor binding.

The results of the KEGG pathway enrichment analysis are illustrated in Fig. 1D, revealing that DE-FRGs are linked to autophagy, ferroptosis, Central carbon metabolism, FoxO signaling pathway, and Lipid metabolism and atherosclerosis. Furthermore, significant enrichment of these differentially expressed ferroptosis-related genes was observed across diverse immune-associated characteristics. To elucidate the function of these genes in distinguishing CRC samples from normal tissues, GSEA-KEGG pathway analysis was performed. Fig. 1E depict the five most enriched pathways for DE-FRGs, highlighting Cytokine-cytokine receptor interaction, Phenylalanine metabolism, NOD-like receptor signaling pathway, Cytosolic DNA-sensing pathway, and Amoebiasis. These observations suggest that DE-FRGs may be instrumental in CRC pathogenesis through their participation in energy metabolism regulation, immune cell activity modulation, and diverse enzymatic activity alterations.

### 3.3 Construction and validation of the machine learning model

**Figure 2:**
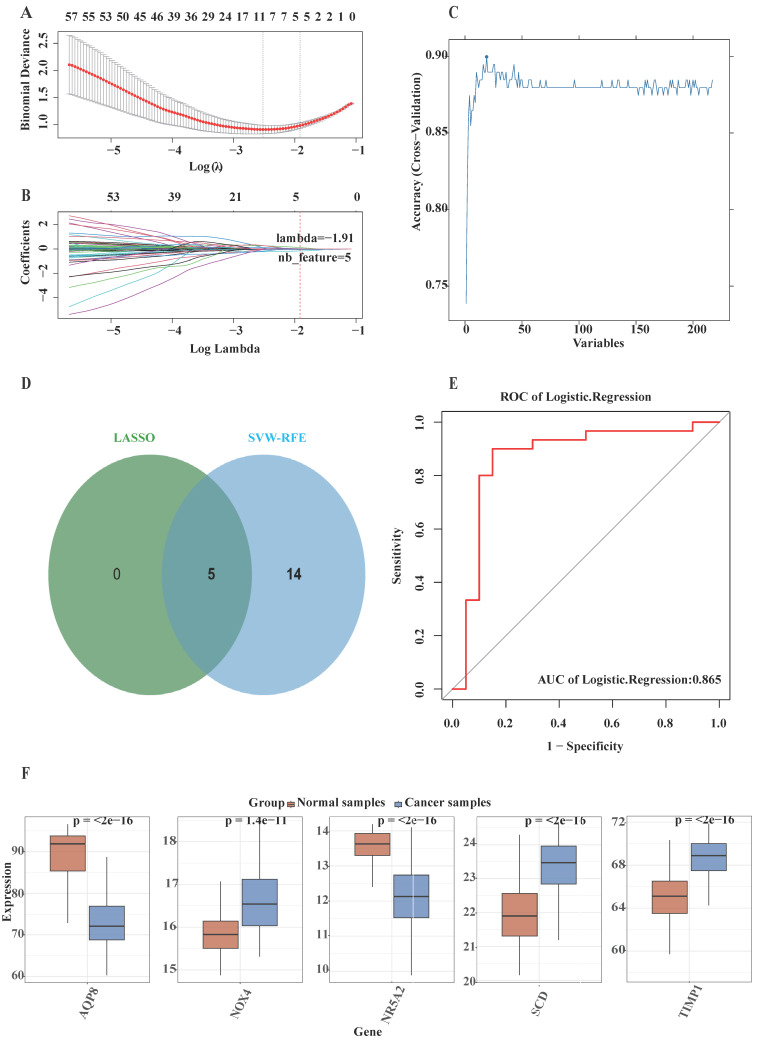
Five DE-FRGs were identified as signature genes for CRC. The specific methodology employed is as follows:** (A and B)** The LASSO logistic regression algorithm was utilized, with penalty parameter tuning performed through 10-fold cross-validation, leading to the selection of 5 genes associated with CRC characteristics; **(C)** The SVW-RFE algorithm was applied to filter the 217 DE-FRGs, determining the optimal combination of feature genes and ultimately identifying 19 genes as the optimal feature set; **(D)** The marker genes obtained from the LASSO and SVW-RFE models are presented; **(E)** The area under the curve (AUC) of the logistic regression model for identifying CRC samples is shown; **(F)** Expression of marker genes in the training cohort.

Based on the observed disparities between CRC patients and healthy individuals, this investigation aimed to evaluate ferroptosis-related potential targets for inhibiting colorectal cancer progression. Two distinct machine learning algorithms—LASSO and SVM-RFE methodologies—were applied to the merged dataset to identify critical DE-FRGs capable of distinguishing CRC patients from normal controls (Table S8). The LASSO logistic regression algorithm with 10-fold cross-validation was employed, resulting in the identification of five candidate genes associated with CRC (Fig. 2A and B). Subsequently, the SVM-RFE algorithm was utilized to further refine the 19 DE-FRGs, thereby identifying the optimal feature combination. Eventually, five genes were established as the most promising potential markers (Fig. 2C). Cross-examination of marker genes selected through two machine learning algorithms highlighted AQP8, NOX4, NR5A2, SCD, and TIMP1 as the principal candidates for succeeding investigation (Fig. 2D). Furthermore, a logistic regression model was constructed using these five potential genes via the R package glm. ROC curve analysis demonstrated that the model effectively discriminated between CRC patients andhealthy individuals, yielding an AUC of 0.865 (Fig. 2E). The logistic regression model exhibited exceptional accuracy and specificity in discriminating ferroptosis-related potential targets for preventing colorectal cancer progression. Following the identification of these five genes, their expression levels were initially analyzed in the merged dataset (Fig. 2F) which demonstrated significantly differential expression patterns between the CRC and normal samples.

### 3.4 External validation of machine learning model

**Figure 3:**
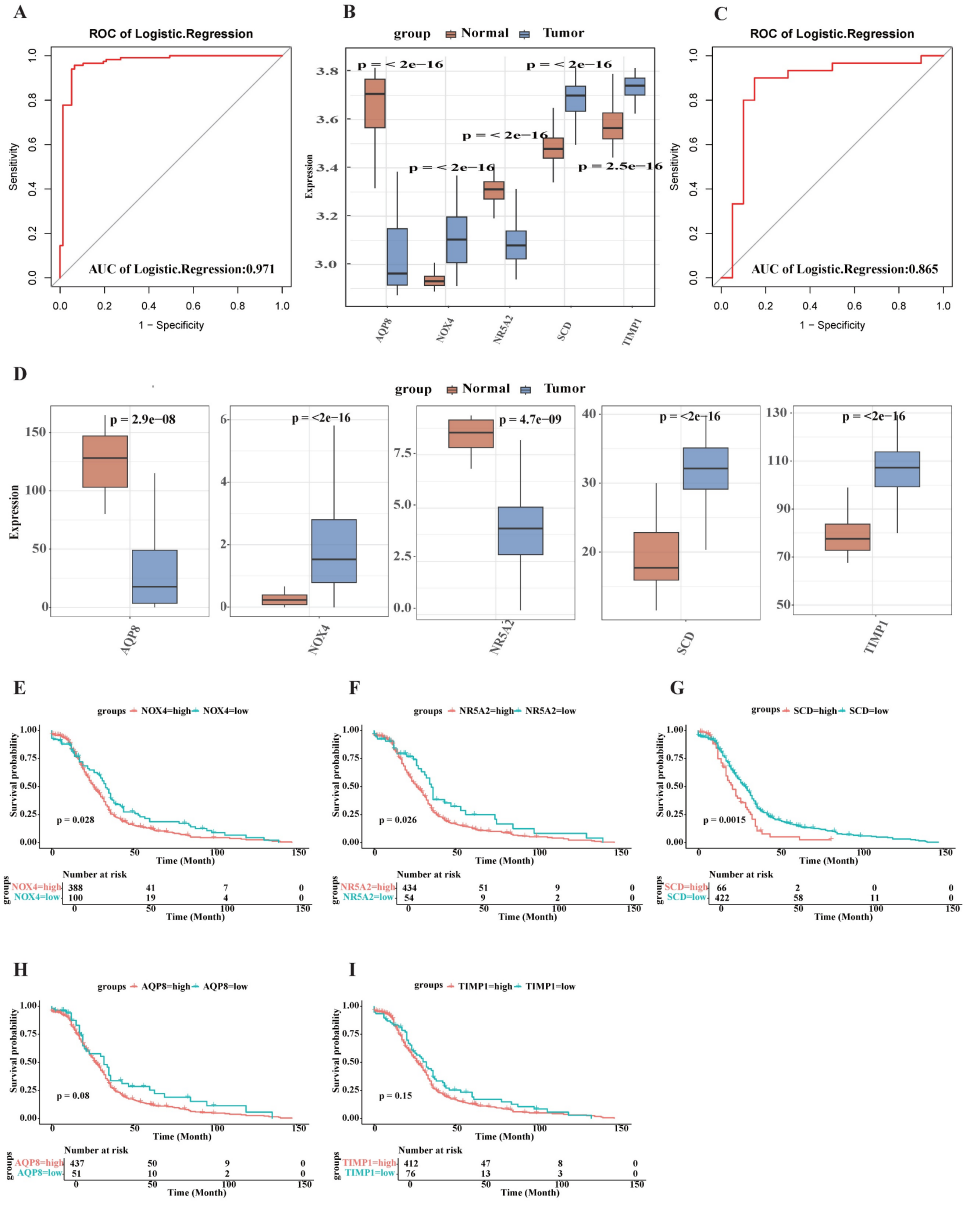
External validation of machine learning model. The specific methodology employed is as follows: **(A)** The area under the curve (AUC) of the logistic regression model for validation cohort (GSE106582); **(B)** Expression of marker genes in the validation cohort (GSE106582);** (C)** The AUC of the logistic regression model for TCGA-COAD cohort; **(D)** Expression of marker genes in the TCGA-COAD cohor;** (F-I)** A study on the effect of five signature genes on patient survival in CRC.

To further evaluate the potential efficacy of the five DE-FRGs, external validation was performed using both GSE106582 and TCGA-COAD datasets through ROC analyses. The AUC value for five DE-FRGs in the GSE106582 cohort was determined to be 0.971 (Fig. 3A). Furthermore, the AUC value in the TCGA-COAD dataset was calculated at 0.865 (Fig. 3C), indicating that these five DE-FRGs possess definite potential values for impeding colorectal cancer progression. The expression levels of these genes were also examined in both GSE106582 (Fig. 3B) and TCGA-COAD datasets (Fig. 3D), revealing significantly differential expression patterns between CRC and normal samples, consistent with observations in the training dataset.

The prognostic significance of the five signature genes in CRC was assessed using the TCGA-COAD dataset for survival analysis, and Kaplan-Meier curves were constructed. As shownin Fig. 3, patients exhibiting elevated expression levels of NOX4 (p < 0.028; Fig. 3E), NR5A2 (p < 0.026; Fig. 3F), and SCD (p < 0.0015; Fig. 3G) demonstrated significantly diminished overall survival compared to those with reduced expression. However, it was observed that NR5A2 expression was higher in normal samples than in CRC samples, with longer overall survival, suggesting that this gene may be susceptible to other influencing factors. No significant associations were detected between the expression of TIMP1 (p = 0.15; Fig. 3H), AQP8 (p = 0.08; Fig. 3I), and overall survival. In conclusion, the analysis identified SCD, and NOX4 as prospective molecular targets with significant potential for CRC prognostication.

### 3.5 GSEA-KEGG pathway analysis of potential targets

To elucidate the functional implications of these potential targets in CRC samples, GSEA-KEGG pathway analysis was conducted. The five most significantly enriched pathways for each potential target have been depicted in Supplementary File 2 Fig. S2-S6. The analytical results revealed that the enriched pathways were associated with diverse biological processes, which include Amino acid metabolism, oxidative phosphorylation, cell cycle regulation and immune response (such as innate immune responses, antigen processing and presentation, Allograft rejection).

### 3.6 DE-FRGs expression profiles in single-cell transcriptome data

**Figure 4:**
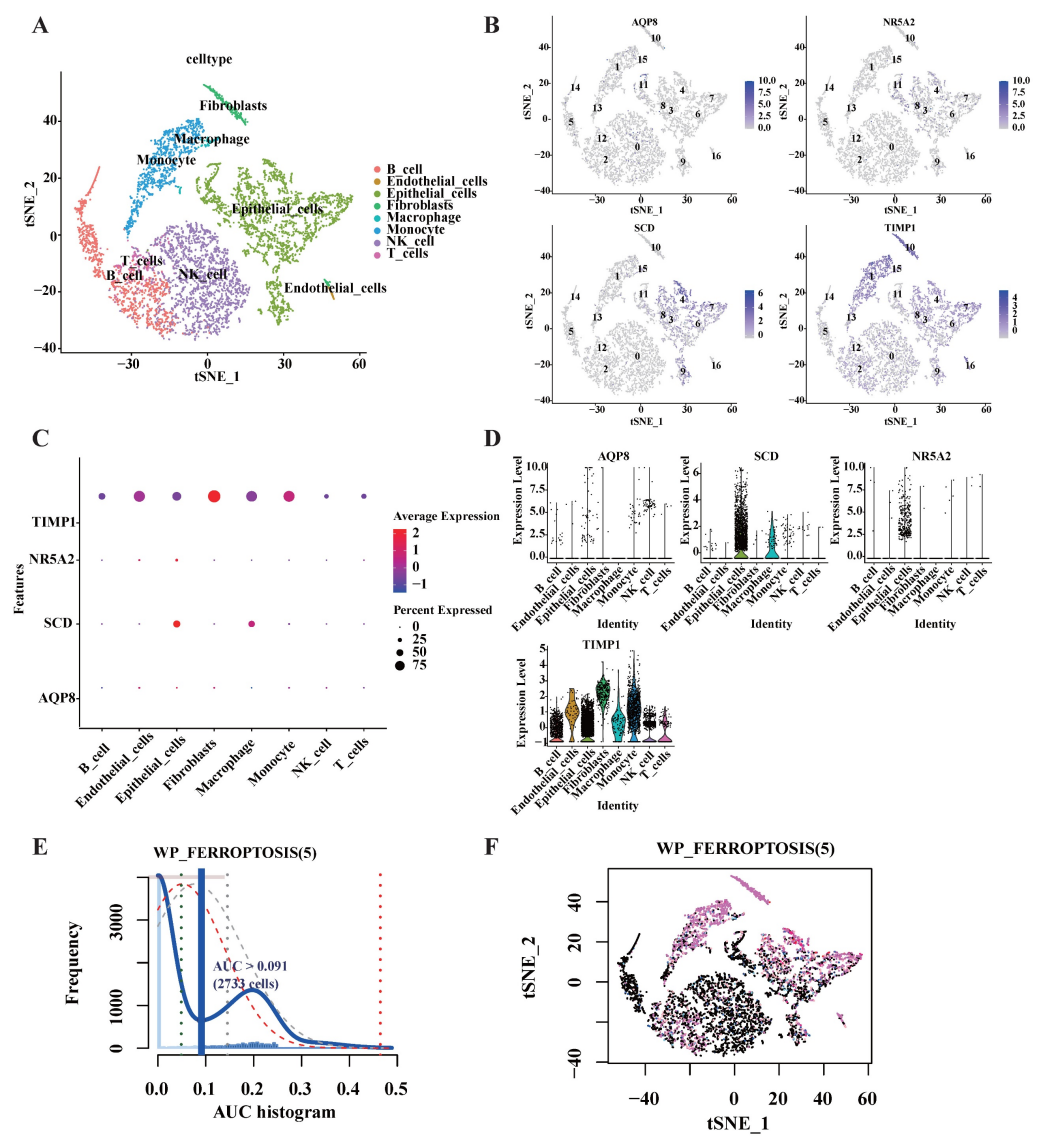
Expression analysis of five signature genes in the single-cell transcriptome dataset GSE161277. **(A)** The t-distributed stochastic neighbor embedding (t-SNE) plot of the eight identified main cell types among single-cell transcriptome dataset GSE161277. **(B)** t-SNE map highlighting the expression of signature genes. **(C)** Bubble plot showing the expression of the signature genes related different cell types. The size of each dot represents the percent expressed; average expression is shown by color. **(D)** Expression of signature genes with eight types of main cell types in single cell transcriptome data. **(E)** Activity profile of the five signature gene (Using the AUCell R package). The threshold value of AUC was 0.091, and 2733 cells exceeded it. (F) The tSNE plot of each cell based on the AUcell score. High-scoring cells are highlighted with red color.

Upon completion of normalization, scaling, clustering, and variable gene filtration processes, dimensionality-reduced clusters from GSE161277 dataset were displayed in a two-dimensional projection. This visualization was achieved by implementing t-distributed Stochastic Neighbor Embedding (t-SNE) techniques subsequent to Principal Component Analysis (PCA), as illustrated in Fig. 4A. Four of the targeted genes were visually represented in cell cluster expression diagrams (Fig. 4B). Notably, NOX4 expression was not detected following data processing procedures. Elevated SCD expression levels were predominantly observed in epithelial cells and macrophages, while TIMP1 expression was detected across all eight identified main cell types within the single-cell transcriptome dataset (Fig. 4C-D).

To characterize the expression patterns of the five targeted genes in CRC, the AUCell R package was employed to determine the five gene activity profile in each cell line (Fig. 4). The AUC values exhibited a bimodal distribution, with 2733 cells demonstrating comparatively higher AUC values above the threshold of 0.091 (Fig. 4E). Cells exhibiting elevated scores were primarily identified as macrophages, epithelial cells, monocyte, fibroblasts, and B cells (Fig. 4F), indicating enhanced activity of the targeted genes in these cellular populations.

### 3.7 The expression of five pivotal ferroptosis-associated gene

**Figure 5:**
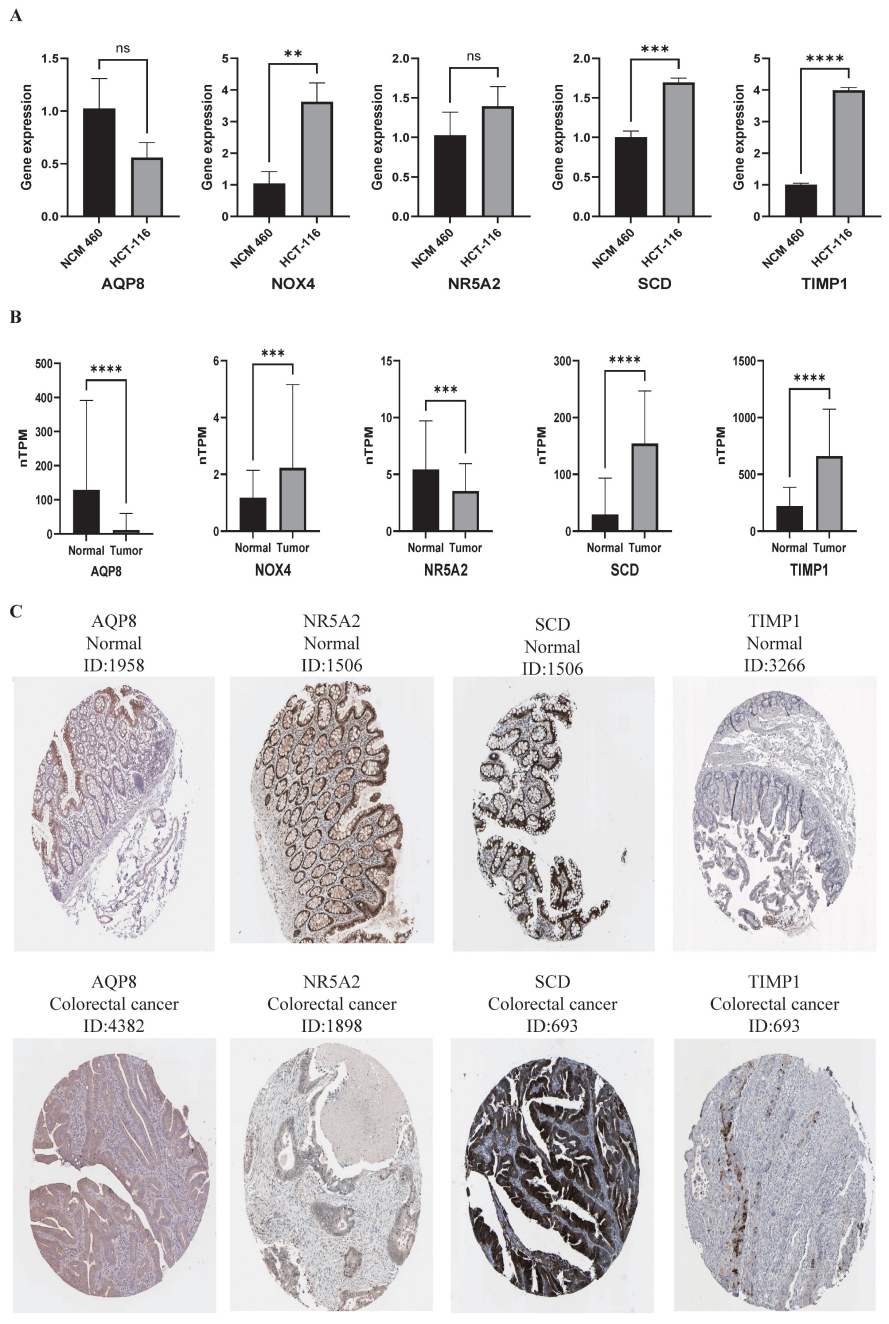
The expression of five pivotal ferroptosis-associated gene. **(A)** The mRNA level of AQP8 was determined by qPCR inCRC and normal colorectal epithelial cell lines. **(B)** The mRNA expression levelsof five key ferroptosis-associated genes from HPA database. **(C)** Immunohistochemistry images of pivotal ferroptosis-associated gene in colorectal cancer and normal tissues detected in the HPA database. ∗P < 0.05, ∗∗P < 0.01, and ∗∗∗P < 0.001.

We quantified the mRNA expression levels of five key ferroptosis-associated genes in both CRC and normal colorectal epithelial cell lines using qRT-PCR to validate the bioinformatic analysis results. Our data showed that the mRNA expression levels of NOX4, SCD, and TIMP1 were significantly higher in CRC cell lines compared with normal colorectal epithelial cell lines (Fig. 5A). However, no significant difference was observed in the relative mRNA expression levels of AQP8 and NR5A2 between the two groups (Fig. 5A). These results indicated that the qRT-PCR validation findings were not entirely consistent with the dataset analysis results, likely due to the use of only a few cell lines (thereby limiting sample representativeness).

To further validate the bioinformatic analysis results, we compared the mRNA expression levels and immunohistochemical (IHC) staining patterns of five key ferroptosis-associated genes between human normal colorectal tissues and colorectal cancer tissues using the HPA database, with an expanded sample size for the comparison. Compared with normal controls, the relative mRNA expression levels of NOX4, SCD, and TIMP1 were significantly higher in tumor tissues, while those of AQP8 and NR5A2 were significantly lower (Fig. 5B). Additionally, we retrieved representative IHC staining images from the HPA database. These images showed that AQP8 and NR5A2 exhibited low staining intensity in colorectal cancer tissues, whereas SCD and TIMP1 showed high staining intensity (Fig. 5C). Unfortunately, no IHC staining data for NOX4 were available in the HPA database. Collectively, these results indicated that the validation findings from the HPA database were consistent with the bioinformatic analysis results.

### 3.8 Immune landscape analysis

**Figure 6:**
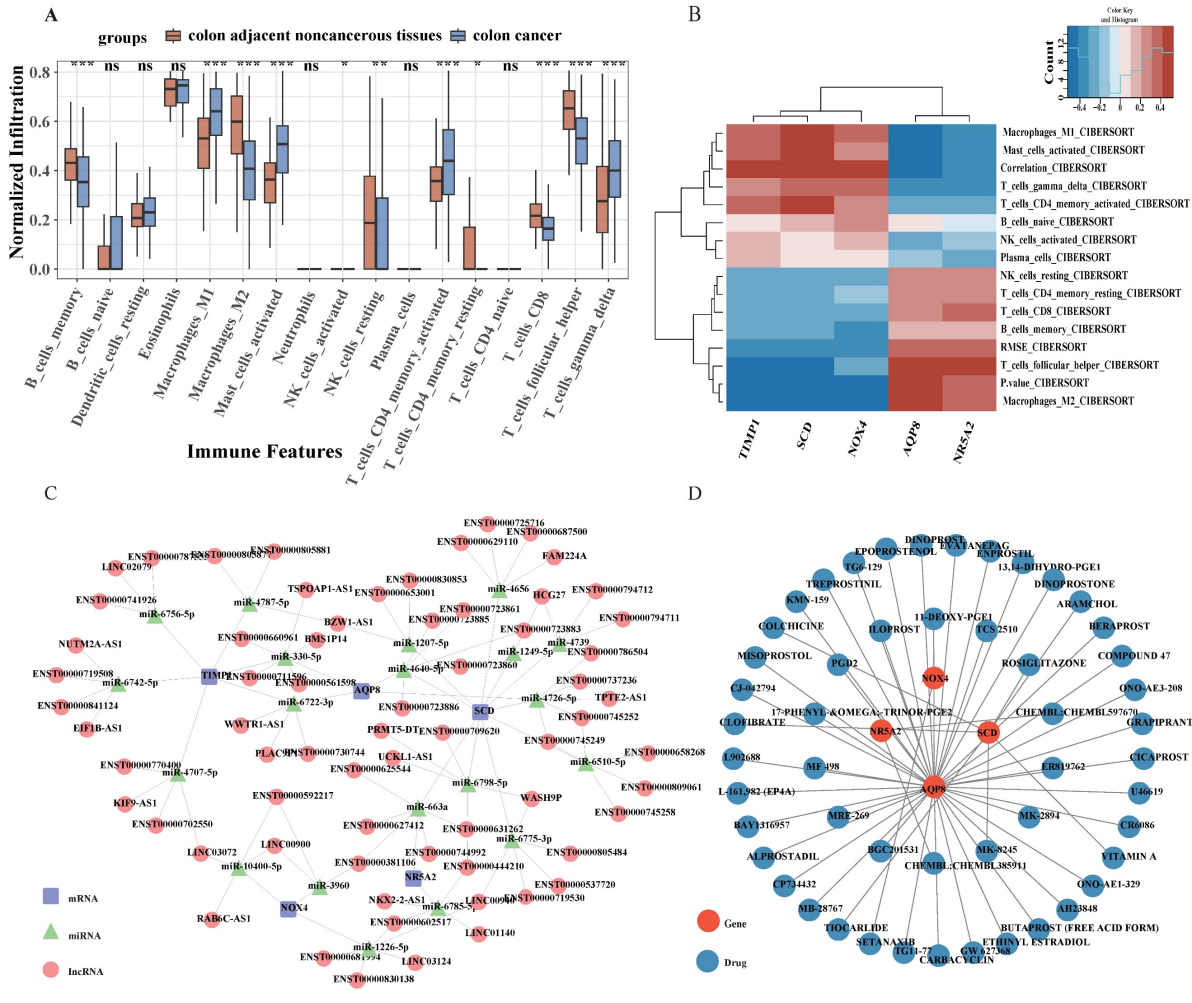
Immune Infiltration Analysis, ceRNA network, and drug network of five signature genes. **(A)**The CIBERSORT algorithm was employed to investigate the differences in the immune microenvironment between CRC patients and normal samples. **(B)** A heatmap illustrating the expression patterns of five signature genes across immune microenvironment. **(C)** The ceRNA network, constructed based on the five marker genes, comprises 571 nodes, which include five marker genes, 20 miRNAs, and 540 lncRNAs, interconnected by 1513 edges (CeRNA network displaying the top five lncRNAs). **(D)** Prediction of targeted drugs for marker genes.

The aforementioned findings suggest a strong association between the identified target genes and immune responses. Moreover, substantial evidence has established an intricate relationship between the immune microenvironment and CRC^26^, ^27^. To examine the immunological differences between CRC and normal samples, the CIBERSORT algorithm was implemented. As presented in Fig. 6A, significantly reduced proportions of memory B cell, M2 Macrophages, resting natural killer NK cells, resting CD4+ memory T lymphocytes, CD8+ T lymphocytes and Follicular helper T cell were observed in CRC patients compared to healthy controls. Conversely, elevated levels of M1 Macrophages, activated mast Cell, activated CD4+ memory T lymphocytes, and gamma delta T cell were detected in CRC samples. Pearson correlation analysis demonstrated that M1 Macrophages exhibited negative correlations with AQP8 and NR5A2, while displaying positive correlations with SCD, NOX4 and TIMP1. Analysis of gamma delta T cells revealed a significant positive association with SCD, NOX4, and TIMP1 expression levels, while simultaneously demonstrating an inverse relationship with AQP8 and NR5A2 expression patterns. CD8+ T lymphocytes positively correlated with AQP8 and NR5A2 (Fig. 6B. These observations suggest that alterations in the immune microenvironment of CRC may be intricately linked to the five identified target genes.

### 3.9 A ceRNA network based on potential targets

A competing endogenous RNA (ceRNA) network was subsequently constructed based on the five identified potential targets, utilizing the miRanda, miRanda, RNAhybrid, and PITA databases. This comprehensive network encompasses 571 nodes (comprising 5 potential targets, 20 miRNAs, and 546 lncRNAs) interconnected by 1513 edges (Fig. 6C). Analysis of the network architecture revealed that 206 distinct lncRNAs canmodulate AQP8 expression through competitive binding with 4 miRNAs, specifically hsa-miR-330-5p, hsa-miR-4640-5p, hsa-miR-4726-5p and hsa-miR-6722-3p. Additionally, 173 lncRNAs were identified to regulate NOX4 expression via competitive binding with 3 miRNAs, including hsa-miR-10400-5p, hsa-miR-1226-5p, and hsa-miR-3960; NR5A2 expression was found to be regulated by 127 lncRNAs through targeting of 2 miRNAs: hsa-miR-663a and hsa-miR-6785-5p. Furthermore, TIMP1 expression appears to be modulated by 372 lncRNAs through competitive binding with 6 miRNAs, such as hsa-miR-330-5p, hsa-miR-4707-5p, and hsa-miR-4787-5p. The most extensively regulated target was SCD, with 635 lncRNAs influencing its expression through interactions with 10 miRNAs, including hsa-miR-1207-5p and hsa-miR-1249-5p. Comprehensive details regarding the ceRNA network are shown in Table S9.

### 3.10 Prediction of potential gene-targeted drugs

Potential therapeutic compounds targeting the five identified genes were investigated utilizing the Drug-Gene Interaction Database (DGIdb) with default parameter settings. The interactions between genes and potential drugs were visualized using Cytoscape software (Fig. 6D). A comprehensive screening revealed 51 compounds that specifically target the potential genes of interest, as detailed in Table S10. Among these, 41 compounds were found to target AQP8, seven compounds targeted SCD, two compounds targeted NR5A2, and one compound targeted NOX4. No targeted therapeutic agents were identified for TIMP1. Furthermore, among the identified compounds, only 11 drugs targeting SCD and AQP8 have received regulatory approval, while no approved drugs currently target TIMP1, NR5A2, or NOX4.

## 4. Discussion

The management of CRC is currently dominated by surgical resection, chemotherapy, targeted therapies, and immunotherapeutic approaches^28^, ^29^. Treatment selection is governed by individual patient's medical condition, tumor characteristics, and American Joint Committee on Cancer staging. Despite standardized staging, CRC exhibits remarkable heterogeneity in prognostic outcomes and therapeutic responses among patients with identical stage classifications^30^. The administration of uniform adjuvant therapies without consideration of genetic and molecular tumor heterogeneity represents a contentious clinical practice. Optimization of therapeutic regimens necessitates the identification of more efficacious prognostic biomarkers and therapeutic targets founded on molecular biology and immunological principles. Ferroptosis, a recently discovered form of regulated cell death, has been documented in large number of malignancies and holds considerable promise for anti-neoplastic therapeutic development^31^, ^32^. This cell death modality may be selectively directed against invasive and aggressive cancer stem cell populations, thereby potentially enhancing immunotherapeutic efficacy and mitigating immunotherapeutic resistance^33^, ^34^. Consequently, ferroptosis-related differentially expressed genes and their encoded proteins may constitute viable anti-cancer interventions for CRC management.

Machine learning, a branch of artificial intelligence that employs statistical algorithms to analyze computational data, is widely applied for selecting features in high-throughput datasets. The combination of bioinformatics and machine learning techniques improves the accuracy and effectiveness of identifying genes associated with diseases, making it a key focus in omics studies. AUCell represents a novel methodological approach enabling the identification of cells with active gene regulatory networks in single-cell RNA-sequencing data, facilitating evaluation of both the proportion of expressed signature genes and their relative expression values compared to the other genes within individual cells^22^. A systematic framework implementing machine learning approaches and AUCell analysis was established in this study to identify core ferroptosis genes and validate their functional association with ferroptosis. Unlike previous studies, which primarily relied on differential expression analysis or single-algorithm screening to identify ferroptosis-related genes in CRC^35^, ^36^, our study applied a combination of machine learning algorithms to screen core genes from differentially expressed genes, followed by cross-validation across multiple datasets. This approach minimizes bias from single-algorithm analysis. More importantly, our study quantified ferroptosis pathway activity at the single-cell level using AUCell, ensuring that the candidate genes are not merely differentially expressed but also closely associated with the functional activity of the ferroptosis pathway. This systematic framework addresses a key limitation of previous studies, which often prioritize genes based solely on expression differences without validating their functional link to ferroptosis.

Meanwhile, bioinformatics and machine learning techniques were employed in the present investigation to elucidate ferroptosis mechanisms in CRC. A robust ferroptosis-related gene signature comprising five genes was established through the combinatorial application of Lasso and SVM-RFE methodologies. The identified signature encompasses several ferroptosis-related genes associated with diverse aspects of cancer development. TIMP1's functionality extends beyond modulation of intracellular oxidative stress levels to potentially influence immune responses, angiogenesis, and tumor cell migration within the tumor microenvironment^37^, ^38^. NOX4 was observed to be significantly enriched in oncogenic-related pathways and demonstrated clear correlations with immune cellular subtypes^39^. SCD functions as a principal enzyme catalyzing the conversion of saturated fatty acidsinto monounsaturated fatty acids. The resultant accumulation of unsaturated fatty acids not only accelerates tumor proliferation and metastasis but also suppresses cell apoptosis and ferroptosis^40^. AQP8, a member of the cell membrane channel protein family, has been demonstrated to influence cellular migration, proliferation, and differentiation processes^41^. Additionally, studies have demonstrated that AQP8 reduces CRC cell proliferation, migration, and invasiveness through the downregulation of PI3K/AKT signaling^42^, ^43^. NR5A2, a transcription factor, regulates the expression of genes specific to certain cell types and tissues. It facilitates tumor growth and metastasis by activating the Wnt/β-catenin signaling pathway^44^. Collectively, these findings illuminate the complex interrelationships between the identified ferroptosis-related genes and their encoded proteins in cancer progression, underscoring their potential utility as therapeutic targets and prognostic indicators in CRC management. Subsequently, cells with active expression of this five-gene ferroptosis-related signature were validated to characterize the expression patterns of the five targeted genes in CRC, using AUCell algorithms. We found that macrophages, epithelial cells, monocyte, fibroblasts, and B cells were most active in activities of five genes. Additionally, the qRT-PCR and THPA database were used to validate the expression of target genes. The result of qRT-PCR showed that NOX4, SCD, and TIMP1 were significantly upregulated, while the expression of AQP8 and NR5A2 did not exhibit the expected differences. The qRT-PCR validation findings were not entirely consistent with the dataset analysis results, likely due to the use of only a few cell lines (thereby limiting sample representativeness). Leveraging the HPA database, we analyzed both IHC staining and mRNA expression in a large number of samples. Both mRNA and IHC analyses from HPA database confirmed the abnormal expression of these pivotal candidate biomarkers. From this, our findings warrant further validation in future studies through the inclusion of large-scale clinical samples and prospective research designs, which will help verify the universality of the identified core ferroptosis genes across different subgroups of CRC.

Our analysis revealed that DE-FRGs play pivotal roles in biological processes including innate immune responses, antigen processing and presentation, and immune response regulation. These findings suggest that these genes might serve as crucial contributors to the pathogenesis and Immune escape mechanisms of colorectal cancer. The results of Gene Set Enrichment Analysis (GSEA) suggested that pathways related to amino acid metabolism and oxidative phosphorylation potentially exert considerable impact on the immune microenvironment surrounding CRC, offering crucial understanding regarding immune cell functions within tumoral contexts. Implementation of the CIBERSORT algorithmic approach revealed notable distinctions in immune microenvironment composition when comparing samples from CRC patients with those from healthy individuals. Several immune cell populations, including CD8+ T lymphocytes, gamma delta T cells, M1 macrophages, and M2 macrophages, demonstrated associations with the five identified ferroptosis-related genes. These observations indicate that changes in the CRC immune microenvironment might be intricately linked to the expression of these target genes. Additionally, utilizing the Drug-Gene Interaction Database (DGIdb), potential therapeutic compounds targeting the signature genes were identified. Notably, only 11 approved drugs were found to target SCD and AQP8, while no approved therapeutic agents currently exist for TIMP1, NR5A2, and NOX4, potentially reflecting limitations in their clinical applicability. Future investigative efforts focused on drug development for these underrepresented genes may establish foundations for novel therapeutic strategies.

While this investigation underscores the pivotal contribution of FRGs in CRC and establishes a novel methodological framework integrating the AUCell algorithm with computational learning techniques, certain constraints warrant recognition. First, the majority of the data analyzed in this study were derived from publicly available databases. Although qRT-PCR validation in cell lines and HPA database analysis partially supported our findings, these results were limited by the small number of cell lines used and the indirect nature of public database validation. Specifically, the lack of large-scale, prospectively collected clinical samples with matched molecular and clinical data restricts the direct generalization of our conclusions to diverse patient populations. Furthermore, while public databases provide valuable resources for large-scale exploratory analyses, they may lack detailed clinical annotations and fail to fully control or explicitly report the detailed inclusion/exclusion criteria of patient cohorts—factors that are critical for deeper translational insights. This limitation restricted our ability to perform stratified analyses on the association between core ferroptosis genes and specific clinical parameters (e.g., tumor stage, differentiation grade, or response to chemotherapy), thereby hindering a comprehensive understanding of their clinical relevance across diverse CRC subgroups. To overcome these limitations, subsequent investigations should be directed toward several critical domains. Initially, original clinical specimens need to be collected for prospective validation. A large cohort of surgically resected CRC tissues (including adjacent normal tissues) with detailed clinical annotations (e.g., patient demographics, treatment history, and survival outcomes) is planned to be collected through collaboration with multi-center clinical institutions. With well-characterized clinical specimens, strict inclusion/exclusion criteria (e.g., no prior anticancer treatment, and complete follow-up data) will be established to ensure cohort homogeneity. This will enable the association between core gene expression and specific clinical subgroups (e.g., early vs. advanced CRC, microsatellite stable vs. unstable tumors) to be explored, and their context-dependent roles in disease progression to be clarified. Subsequently, functional experiments with clinical data were integrated. Future work will combine in vitro functional assays (e.g., gene knockout/overexpression in CRC cell lines, ferroptosis induction assays) with in vivo animal models to validate the mechanistic roles of core genes in regulating ferroptosis. These functional data will be integrated with clinical sample analysis to determine whether these genes can serve as predictive biomarkers for ferroptosis-targeted therapies or prognostic indicators in CRC.

Distinct from previous prognostic models^35^, ^45^, Our investigation has employed a novel methodological framework that integrates the AUCell algorithm with computational learning techniques, thereby characterizing five prospective targets among ferroptosis-related genes in CRC patients. This framework prioritizes genes based on expression differences with validating their functional link to ferroptosis. By integrating AUCell-based functional validation with machine learning algorithms and leveraging multiple databases, including GEO, HPA, and TCGA, we not only expanded the sample size for analyzing ferroptosis-related core genes in CRC but also rigorously validated the universality of these ferroptosis-related core genes across CRC contexts. This approach establishes opportunities for the advancement of innovative therapeutic interventions. The modulation or inhibition of ferroptosis may be achieved through targeting these genes or biological pathways within the FRG signature, potentially leading to enhanced treatment strategies for CRC. Such approaches could encompass the development of pharmaceutical interventions that combine conventional chemotherapy with small-molecule inhibitors or selective agents that target ferroptosis-related pathways. Furthermore, the integration of these FRGs and associated pathways with additional omics data, including epigenomics and metabolomics, could potentially unveil novel biomarkers and therapeutic targets. Through the amalgamation of multiple strata of molecular data, a comprehensive understanding of the intricate interactions between various biological processes can be gained, enabling the identification of key molecular entities suitable for therapeutic intervention.

## 5. Conclusion

In summary, A systematic framework implementing machine-learning approaches and AUcell analysis was established in this study to identify core ferroptosis genes and validate their functional link to ferroptosis. Meanwhile, a robust and reliable ferroptosis-related signature comprising AQP8, NOX4, NR5A2, SCD, and TIMP1 was established by analyzing the sequencing data of ferroptosis-related differentially expressed genes in CRC. The efficacy of this signature has been substantiated across multiple independent datasets, with further validation of its relationship to immune cell infiltration. These findings offer fresh insights into the ferroptosis-associated molecular mechanisms underlying CRC, with particular emphasis on the importance of this ferroptosis-related signature within the immune microenvironment and potential therapeutic interventions targeting these genes—aspects that merit additional exploration. Future research efforts should prioritize validating the functional characteristics of this ferroptosis-related signature and clarifying its interactions with immune cell components, thereby unveiling innovative approaches for managing CRC.

## Supplementary Material

Supplementary tables.

Supplementary figures.

## Abbreviations

AUC: area under the curve
CRC: colorectal cancer
DE-FRGs: differentially expressed ferroptosis-related genes
DGIdb: drug-gene interaction database
FRGs: ferroptosis-related genes
GEO: gene expression omnibus
GO: gene ontology
GSEA: gene set enrichment analysis
HPA: Human Protein Atlas
IHC: immunohistochemistry
KEGG: kyoto encyclopedia of genes and genomes
LASSO: least absolute shrinkage and selection operator
qRT-PCR: perform quantitative real-time reverse transcription PCR
ROC: receiver operating characteristic
SVM-RFE: support vector machine recursive feature elimination
TCGA: the cancer genome atlas
TCGA-COAD: the cancer genome atlas colon adenocarcinoma data
