# Supplementary material for: Identification of key ferroptosis-related targets in colorectal cancer: A transcriptomics-driven study via machine learning and AUcell analysis of single-cell RNA-sequencing
Source: J Cancer. 2026 Jan 1;17(1):32–48. doi: 10.7150/jca.114522 (PMC12719592; doi:10.7150/jca.114522)
Supplement: Supplementary file 2 — Supplementary figures. [file jcav17p0032s2.pdf]

Identification of key ferroptosis-related targets in colorectal cancer: A multiomics study via machine learning and AUcell analysis of single-cell RNA-sequencing

ZhiqiangLiang[1,†], ZehuiHou[1,†], ZhuominYu[1,†], BingZeng[1], FangLi[2], JingjingWu[2], YingruLi[1,*],ZhipengJiang[2,*]

[1]Department of General Surgery, Hernia and Abdominal Wall Surgery, The Sixth Affiliated Hospital, Sun Yat-sen University,Guangzhou City510655, Guangdong Province, China

[2]Division of Gastrointestinal Surgery,Department of General Surgery, Shenzhen People' s Hospital(The Second Clinical Medical College, Jinan University; The First Affiliated Hospital, Southern University of Science and Technology), Shenzhen 518020, Guangdong Province, China

* Corresponding author: E-mail address: jzpsums@126.com(Z. Jiang); liyingru@mail.sysu.edu.cn(Y. LI);

Fig. S1. Flow chart of this study

Fig. S2.The results of Gene Set Enrichment Analysis - Kyoto Encyclopedia of Genes and Genomes (GSEA-KEGG) pathway analyses for the AQP8 protein.

Fig. S3.The results of Gene Set Enrichment Analysis - Kyoto Encyclopedia of Genes and Genomes (GSEA-KEGG) pathway analyses for the NOX4 protein.

Fig. S4.The results of Gene Set Enrichment Analysis - Kyoto Encyclopedia of Genes and Genomes (GSEA-KEGG) pathway analyses for the NR5A2 protein.

Fig. S5.The results of Gene Set Enrichment Analysis - Kyoto Encyclopedia of Genes and Genomes (GSEA-KEGG) pathway analyses for the SCD protein .

Fig. S6.The results of Gene Set Enrichment Analysis - Kyoto Encyclopedia of Genes and Genomes (GSEA-KEGG) pathway analyses for the TIMP1protein.

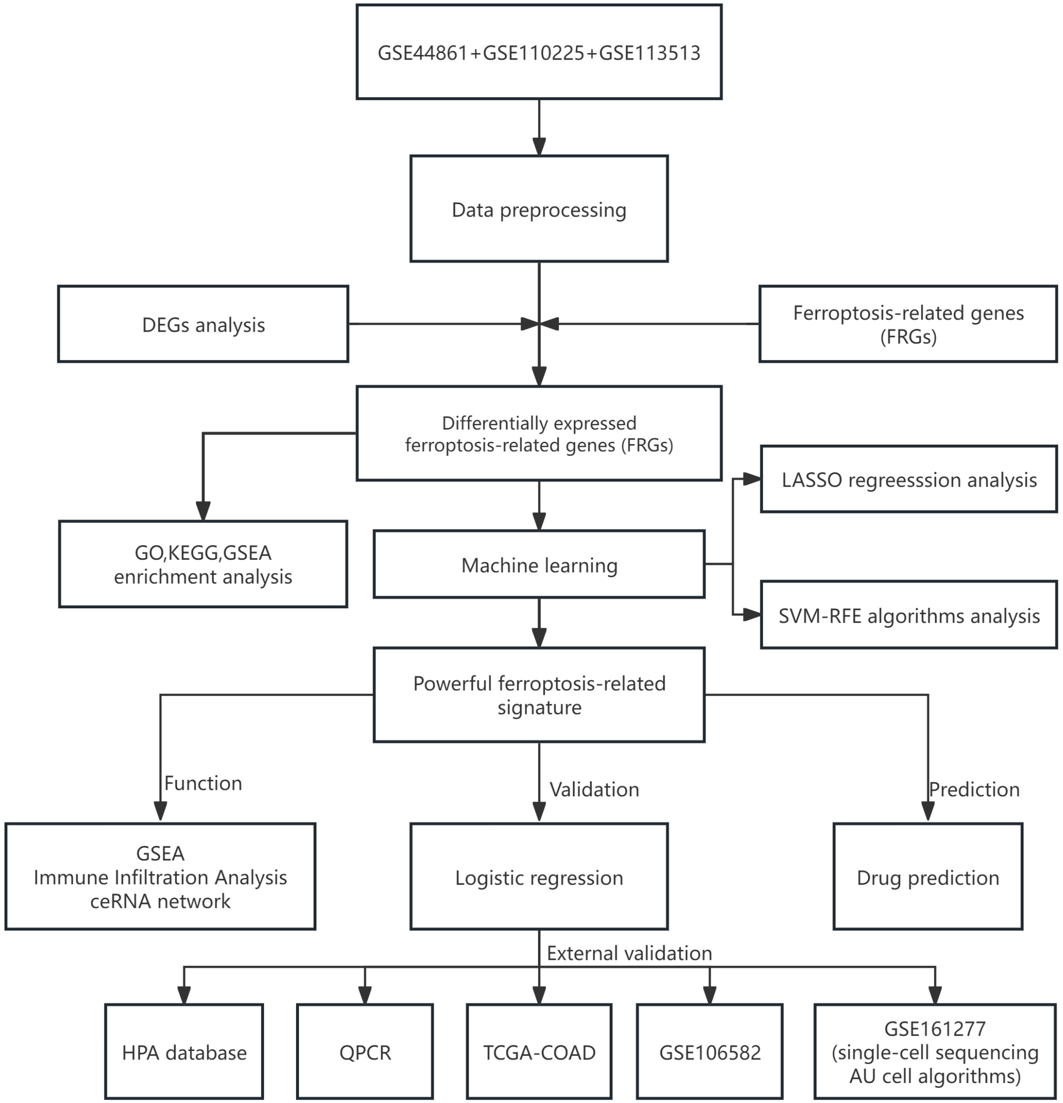

Fig. S1. Flow chart of this study

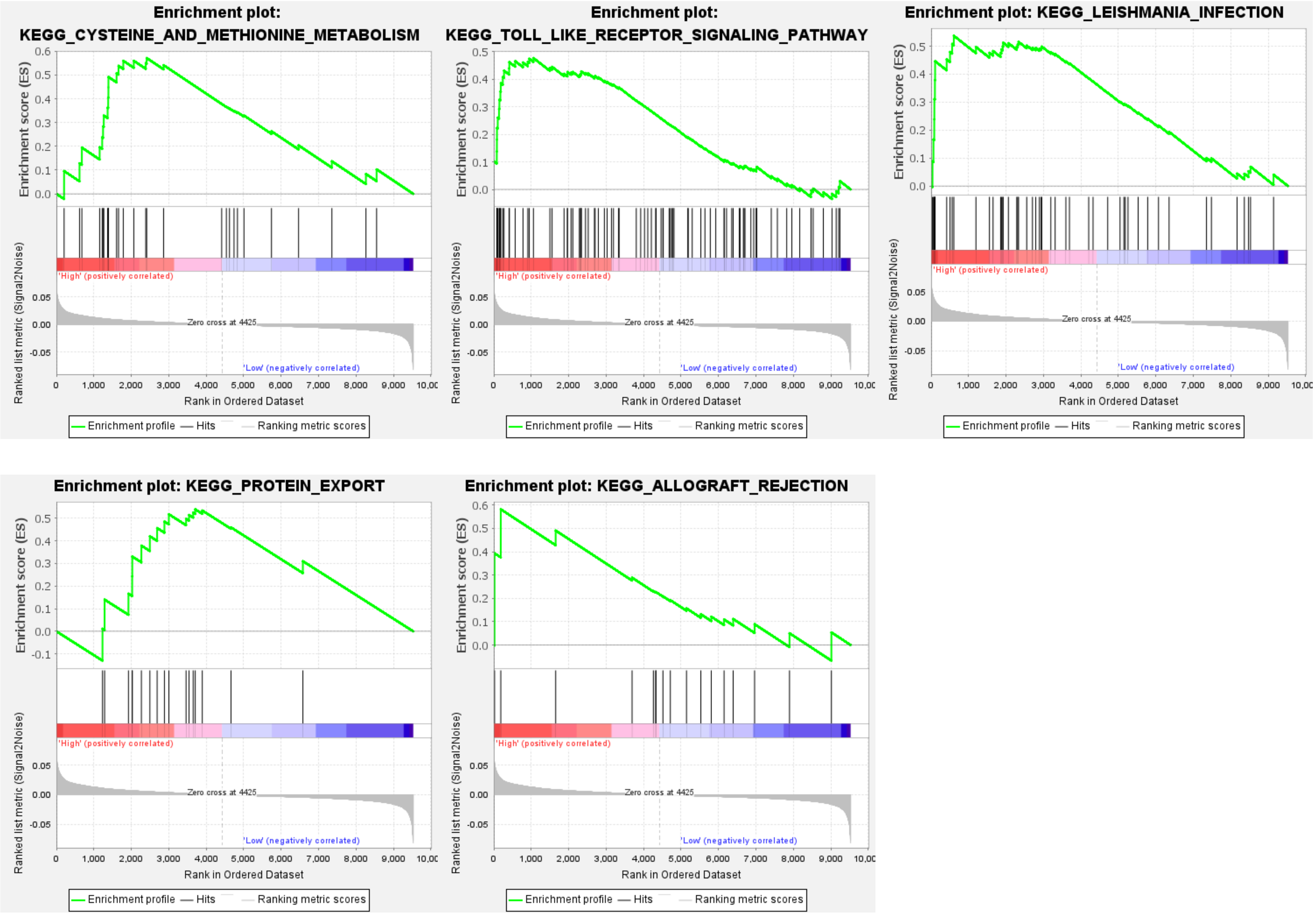

Fig. S2.The results of Gene Set Enrichment Analysis - Kyoto Encyclopedia of Genes and Genomes (GSEA-KEGG) pathway analyses for the AQP8 protein (An examination of the top five routes using).

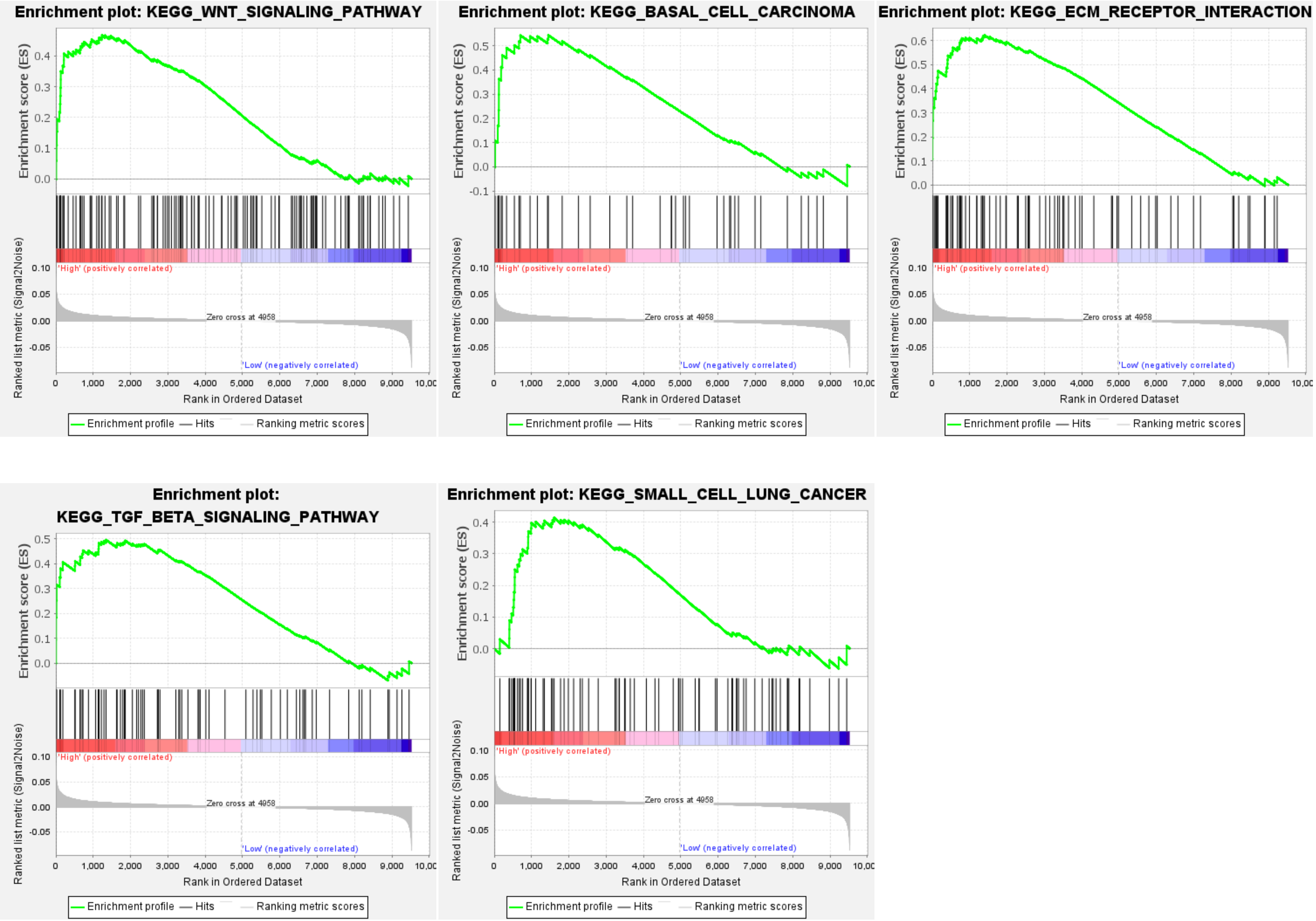

Fig. S3.The results of Gene Set Enrichment Analysis - Kyoto Encyclopedia of Genes and Genomes (GSEA-KEGG) pathway analyses for the NOX4 protein (An examination of the top five routes using).

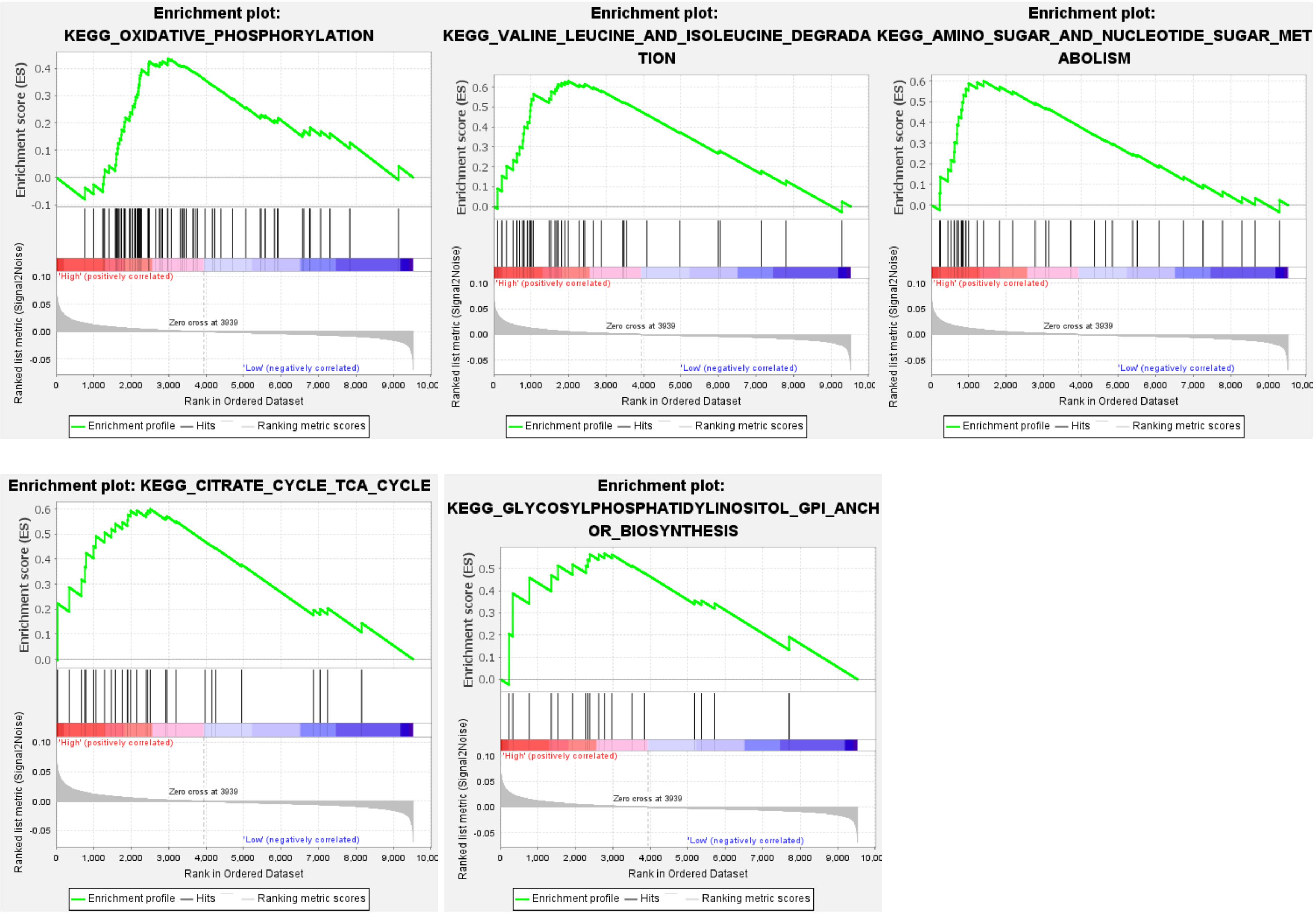

Fig. S4.The results of Gene Set Enrichment Analysis - Kyoto Encyclopedia of Genes and Genomes (GSEA-KEGG) pathway analyses for the NR5A2 protein (An examination of the top five routes using).

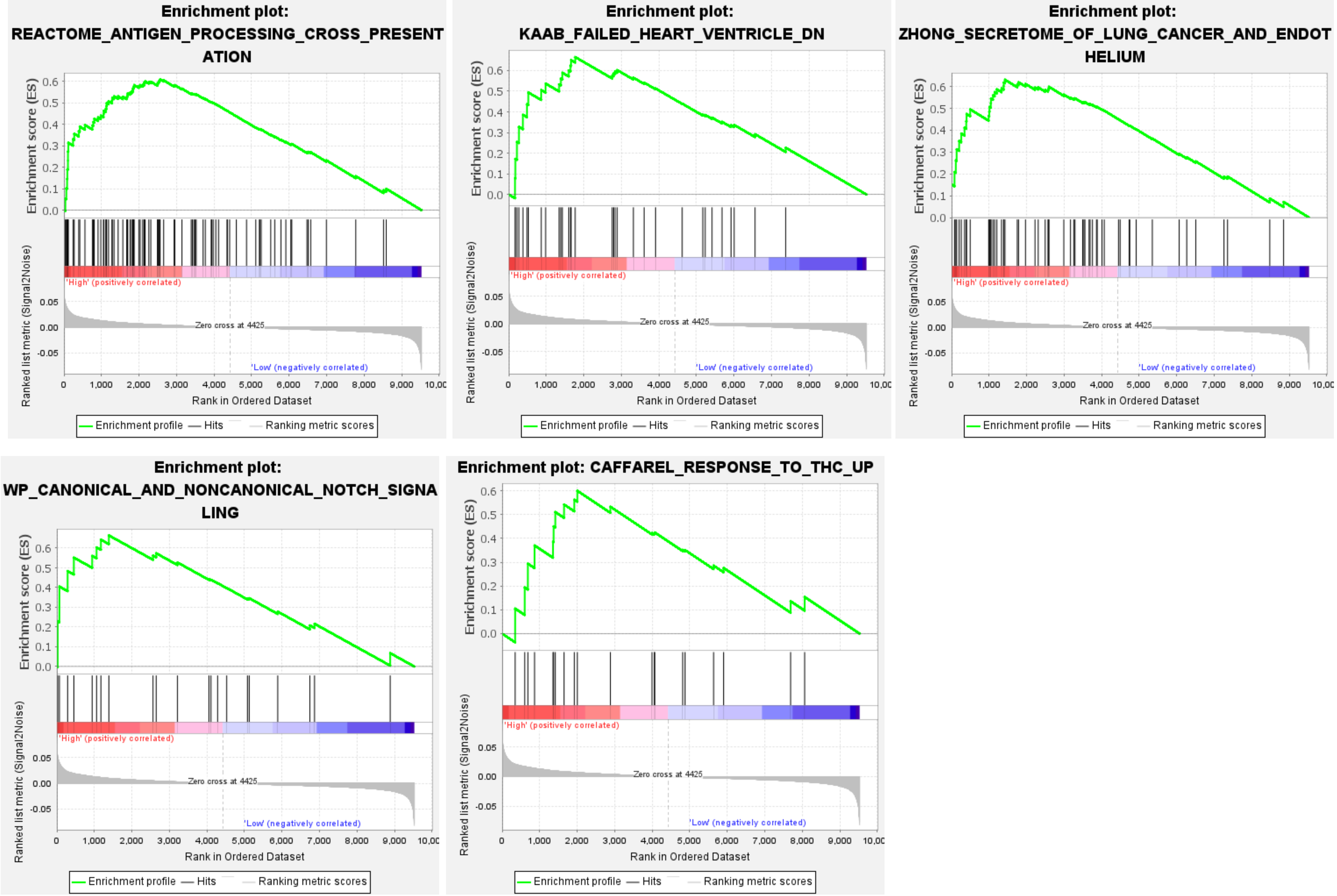

Fig. S5.The results of Gene Set Enrichment Analysis - Kyoto Encyclopedia of Genes and Genomes (GSEA-KEGG) pathway analyses for the SCD protein (An examination of the top five routes using).

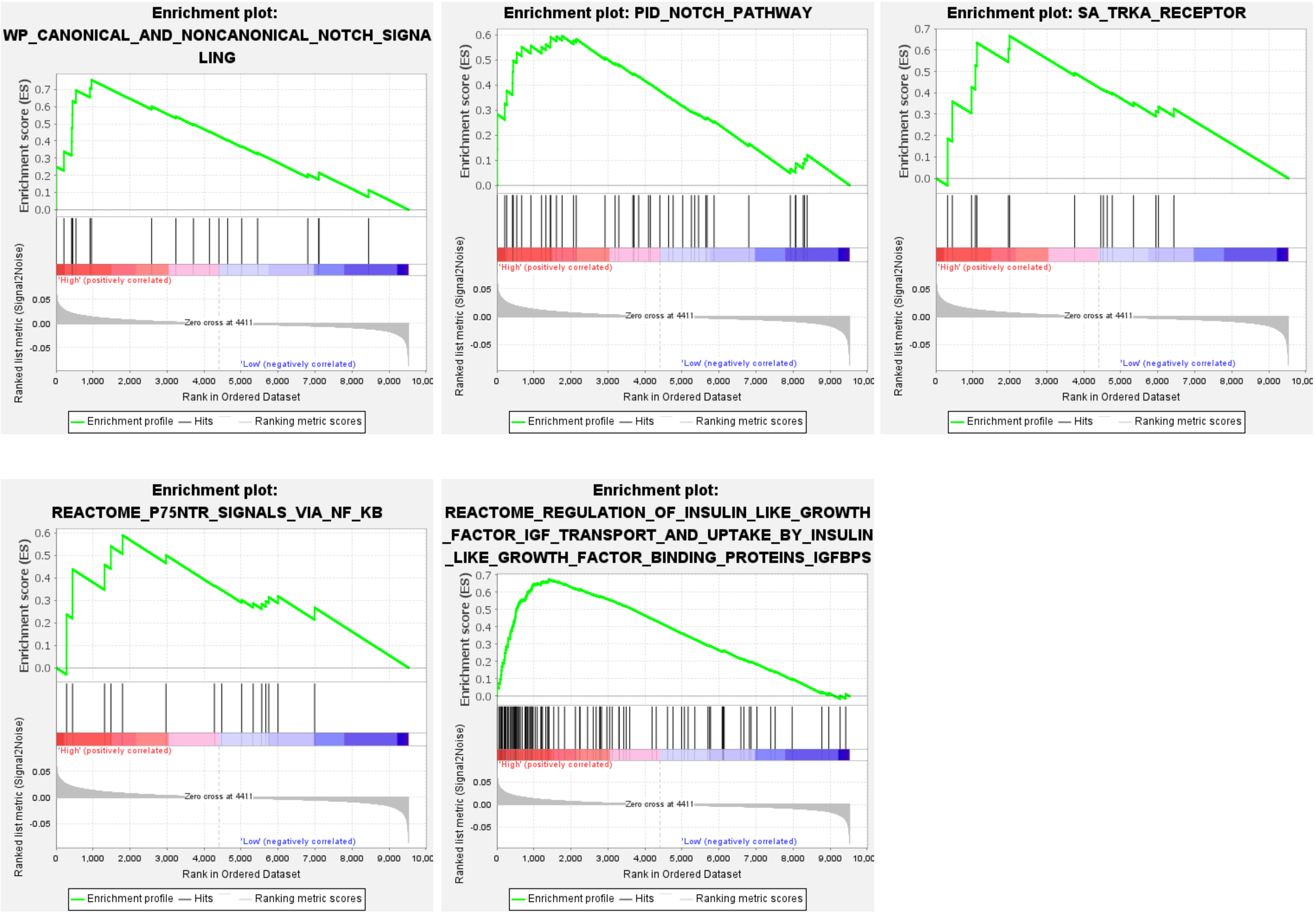

Fig. S6.The results of Gene Set Enrichment Analysis - Kyoto Encyclopedia of Genes and Genomes (GSEA-KEGG) pathway analyses for the TIMP1protein (An examination of the top five routes using).
